# An mHealth Management Platform for Patients with Chronic Obstructive Pulmonary Disease (efil breath): Randomized Controlled Trial

**DOI:** 10.2196/10502

**Published:** 2018-08-24

**Authors:** Hee Kwon, Sungin Lee, Eun Ji Jung, SangHee Kim, Jung-Kyu Lee, Deog Kyeom Kim, Tae-Hyung Kim, Seung Hyeun Lee, Myoung Kyu Lee, Seungjae Song, Kichul Shin

**Affiliations:** ^1^ LifeSemantics Corp Seoul Republic Of Korea; ^2^ Seoul Metropolitan Government-Seoul National University Boramae Medical Center Seoul Republic Of Korea; ^3^ Department of Internal Medicine Seoul Metropolitan Government-Seoul National University Boramae Medical Center Seoul Republic Of Korea; ^4^ Division of Pulmonology Hanyang University College of Medicine Gyeonggi-do Republic Of Korea; ^5^ Department of Internal Medicine Kyung Hee University School of Medicine Seoul Republic Of Korea; ^6^ Department of Internal Medicine Yonsei University Wonju College of Medicine Gangwon-do Republic Of Korea

**Keywords:** chronic obstructive pulmonary disease, mHealth, mobile phone, physical activity, rehabilitation, quality of life

## Abstract

**Background:**

Chronic obstructive pulmonary disease (COPD) is one of the major morbidities in public health, and the use of mHealth technology for rehabilitation of patients with COPD can help increase physical activity and ameliorate respiratory symptoms.

**Objective:**

This study aimed to develop a comprehensive rehabilitation management platform to improve physical activity and quality of life in patients with COPD.

**Methods:**

The study comprised the following 2 stages: (1) a pilot stage in which a prototype app was developed; and (2) a fully-fledged platform development stage in which 2 apps and 1 COPD patient monitoring website were developed. We conducted a randomized clinical trial to investigate the efficacy of the apps developed in the second stage of the study. In addition, two 12-week exercise regimens (fixed and fixed-interactive) were tested for the trial. The clinical parameters of the respiratory function and patient global assessment (PGA) of the app were obtained and analyzed. Notably, Android was the chosen operating system for apps.

**Results:**

We developed 2 COPD rehabilitation apps and 1 patient monitoring website. For the clinical trial, 85 patients were randomized into the following 3 groups: 57 were allocated to the 2 intervention groups and 28 to the control group. After 6 weeks, the COPD assessment test scores were significantly reduced in the fixed group (*P*=.01), and signs of improvement were witnessed in the fixed-interactive group. In addition, the PGA score was moderate or high in all aspects of the user experience of the apps in both intervention groups.

**Conclusions:**

A well-designed mobile rehabilitation app for monitoring and managing patients with COPD can supplement or replace traditional center-based rehabilitation programs and achieve improved patient health outcomes.

**Trial Registration:**

ClinicalTrials.gov NCT03432117; https://clinicaltrials.gov/ct2/show/NCT03432117 (Archived by WebCite at http://www.webcitation.org/71Yp0P64a)

## Introduction

Chronic obstructive pulmonary disease (COPD) is recognized as a major public health problem and might become a considerable burden worldwide in the near future [1]. The same phenomenon has been witnessed in Korea, where COPD has become the sixth leading cause of death, and its prevalence has reached close to 13% among individuals aged ≥40 years (19.4% of males and 7.9% of females) [2,3]. Pulmonary rehabilitation (PR) is a comprehensive intervention through which patient assessment, exercise training, education, nutritional intervention, and psychosocial support [4,5] are administered to meet the goals of improved physical and psychological condition, for example, exercise capacity and quality of life (QoL), and reduced health care utilization [6]. However, it is challenging to ensure that patients with COPD do conform to the recommended and agreed-upon quantity and quality of rehabilitation programs as part of their disease management plan [7], and patients with severe or extreme disease activity tend to exhibit fewer and shorter bouts of physical activity [8,9]. Furthermore, the factors affecting low uptake and incompletion of PR include the low degree of perceived benefits and the lack of support for transport in these patients [7].

According to a 2016 survey of 7 tertiary hospitals in Korea that provided PR programs, only 5 hospitals had established protocols for PR programs, while 2 hospitals had only conventional rehabilitation programs. Inpatients were admitted to a 1- to 2-week PR program with an average of 3-5 sessions a week, and each session ran 10-60 minutes. Only 1 hospital had a 12-week PR program with exercise programs and patient education sessions focusing on the muscular endurance, cardiorespiratory fitness, and breathing training. Though most of the hospitals’ survey acknowledged the need for an extended PR program, the hardships included a lack of funding or certified facilities, and low national health insurance coverage. An alternative model that assists in overcoming these barriers is home-based rehabilitation (HBR) [10,11]. A well-structured HBR has the potential to surpass center-based rehabilitation by promoting exercise capacity and health-related QoL [12-14]. Many established HBR programs yet require qualified health care professionals, such as physiotherapists or home-care nurses, who periodically pay a visit to patients [10,12]. Without tracking physical activity automatically [15], the burden of manual entry of the vast amount of data, such as exercise duration and walked distance [13], lies on health care professionals and patients. The burden of data recording, for both patients and health care professionals, could be solved by accessible, user-friendly mHealth technology [16,17]. Equipped with mobile apps and monitoring platforms that can manage COPD patients’ PR, health care professionals are better positioned to monitor patients’ compliance and activities and provide accurate feedback.

A significant body of studies exists proving that HBR using mobile technology is as effective as center-based rehabilitation programs [15,18]. Specifically, home-based COPD rehabilitation programs have been an optimal alternative to center-based rehabilitation; improved exercise capacity and QoL resulted in increased physical activity and reduced respiratory-related hospitalizations [10,13,19-23]. Mobile technology assists automatic data recording of exercise activities, and certain apps send data to a central server, where health care professionals with proper clearance use the data for patient monitoring and feedback.

There are, however, only a few available HBR mobile apps that incorporate evidence-based health recommendations [24]. To achieve better health outcomes in patients with COPD, the following requirements for mobile HBR programs should be met: (1) exercise programs must conform to evidence, such as public health recommendations (ie, the Consensus Document on Pulmonary Rehabilitation in Korea) [24]; (2) a baseline assessment of exercise capacity, such as the 6-minute walk test (6MWT), must be provided prior to the onset and end of the exercise [25]; (3) exercise regimens should be adequately flexible to be adjusted according to the patient status [4]; and (4) a patient management and monitoring platform should be present [26].

This study aimed to develop a home-based mHealth PR for patients with COPD to improve their daily physical capacity and QoL. To achieve this goal, we developed *efil breath*, which combines a mobile PR app platform, including a wearable device, a personalized app, and a website for monitoring patients by health care professionals. Furthermore, a randomized clinical trial was conducted to investigate the effectiveness of the platform. Notably, the study is the first multicenter-based clinical trial of a home-based mobile PR program for Korean patients with COPD.

## Methods

### Study Design

**Figure 1:**
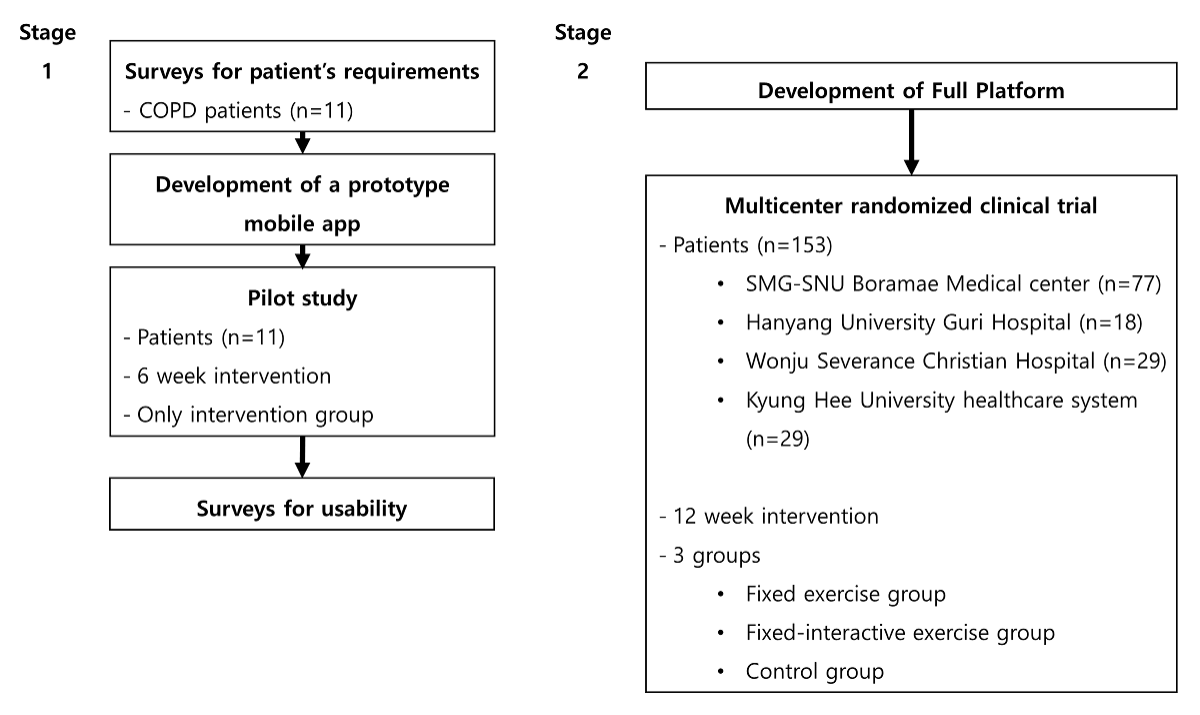
Study design. COPD: Chronic obstructive pulmonary disease; SMG-SNU: Seoul Metropolitan Government-Seoul National University.

The study was divided into the following 2 stages (Figure 1): a pilot study (Stage 1), and the full development of a platform, followed by a clinical trial (Stage 2). Stage 1 consisted of the following 4 steps: (1) collection of user requirements using survey results from patients with COPD (n=11), who used the home-based PR, and in-person consultations obtained from qualified health care professionals; (2) development of a prototype mobile app; (3) a 6-week pilot study testing the app (no control group) to assess the study feasibility; and (4) a usability survey. Stage 2 consisted of (1) the development of 2 types of mobile apps (one with a fixed exercise regimen, and another with an interactive exercise regimen) and a patient management or monitoring website; and (2) a 12-week, multicenter-based randomized clinical trial. The trial participants in the intervention groups were instructed to use the app with a fixed or interactive exercise regimen, and those in the control group went on with their daily lives without using the app.

### Participants of the Clinical Trial

The study participants of Stage 2 were recruited from outpatient clinics of 4 secondary or tertiary hospitals in Korea. Patients with COPD were selected according to the following inclusion criteria: (1) age>20 years; (2) a postbronchodilator forced expiratory volume in 1 second of <80% compared with the reference range; (3) ability to walk >150 m in a 6MWT; and (4) an Android smartphone owner. Of note, patients who were unable to follow the exercise regimen were excluded from the screening process. All study participants were to sign a written informed consent.

### Clinical Trial Protocol

The study participants were randomized into 3 groups as follows: fixed exercise, fixed-interactive exercise, and control group. The fixed-interactive exercise group initiated a fixed exercise regimen for the first 6 weeks, followed by an interactive exercise protocol for 6 weeks. A random allocation (1:1:1) within each center was moderated by an independent coordinator; patients were stratified by the baseline forced expiratory volume in 1 second and COPD assessment test (CAT) scores. The 6MWT, self-perceived dyspnea assessment in relation to a physical disability (modified Medical Research Council, mMRC), and CAT were acquired at the baseline (V1), 6 weeks (V2), and 12 weeks (V3). Patient global assessment (PGA) by a 5-point Likert scale (1=strongly disagree, 2=disagree, 3=neither disagree nor agree, 4=agree, 5=strongly agree) was measured at V3. Participants in both intervention groups were provided with a wearable pulse oximeter (Checkme O2, Viatom, China). The trial commenced in May 2017 and ended in December 2017 and was approved by the Institutional Review Board of each participating hospital. In this study, the primary endpoint was the change of respiratory function parameters (6MWT, CAT, and mMRC) at V3 compared with the baseline.

### Statistical Analysis

All statistical analyses were performed using SPSS, version 18.0 (IBM, Armonk, New York, United States). One-way analysis of variance was used to compare the baseline characteristics of the 3 groups. In addition, one-way analysis of variance with repeated measures was performed to analyze changes between visits. We considered *P<*.05 as a statistically significant difference. Based on previous studies showing 6MWT improvement in clinical trials, we hypothesized a mean difference of 6WMT to be >50 m in the intervention group versus 0 m in the control group after 12 weeks [27,28]. Assuming an SD of 60, a two-sided test of an alpha level of.05, a power of 80%, and a participant dropout rate of 20%, a sample size of 84 patients (28 per group) was required for the primary analysis.

## Results

### Pilot Study (Stage 1)

#### User Requirements for Mobile App and Wearable Device

To collect user requirements for the home-based COPD rehabilitation app and wearable devices, we recruited 11 patients (7 males and 4 females) for the interview. Textbox 1 describes the final user requirements.

In tandem with patient interviews, a comprehensive literature review and in-person consultations were obtained from health care professionals regarding the requirements for the app, wearable devices, and the patient monitoring website. In addition, patients were asked to perform a 30-minute walk every day, and blood oxygen saturation (SpO_2_) and heart rate were measured and displayed to users during exercise.

#### Usability Evaluation

**Figure 2:**
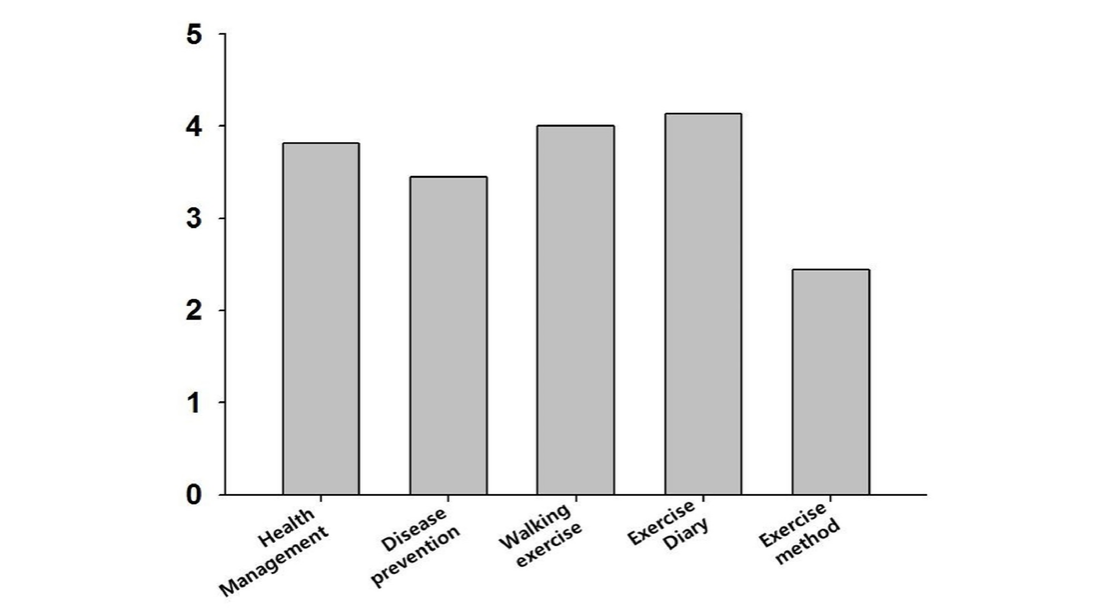
Usability evaluation results (Stage 1).

After 6 weeks of use, a simple usability test was performed. Of initial 12 participants, 11 participants completed a 5-item usability questionnaire using a 5-point Likert scale. The results showed that the “Exercise Diary” was the highest and “Exercise Method” the lowest (Figure 2). The average score was 3.56.

### Stage 2

**Figure 3:**
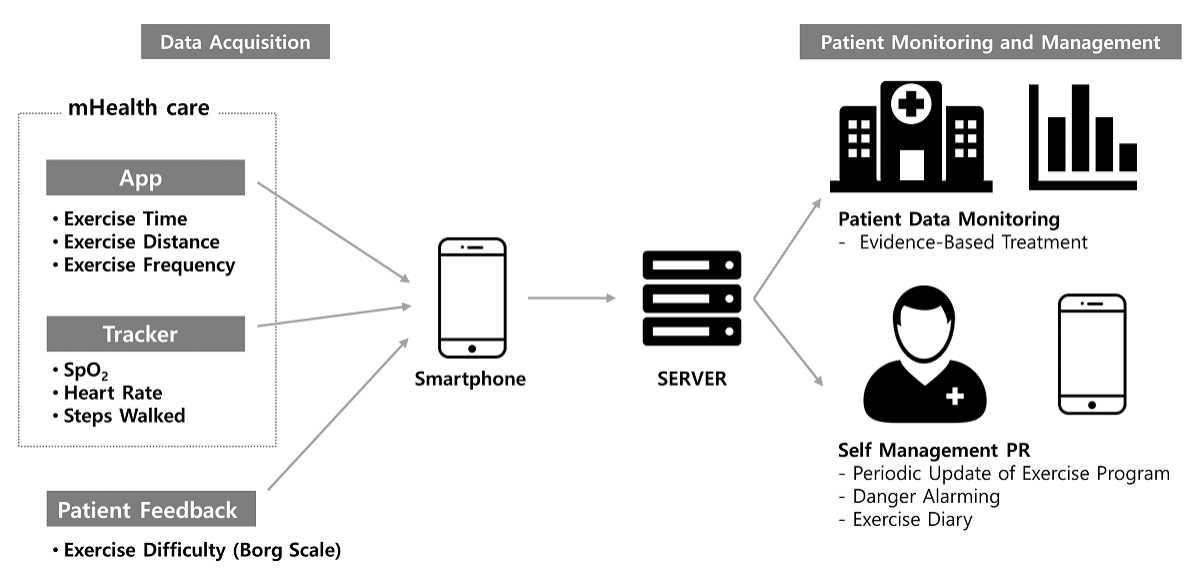
Architecture of the home-based mobile COPD care service. PR: pulmonary rehabilitation; SpO2: blood oxygen saturation.

Figure 3 shows the main components of our platform, *efil breath*, developed in Stage 2; it includes 1 wearable pulse oximeter, 2 mobile apps, and 1 patient monitoring website. The apps access time, distance and frequency of the exercise, heart rate, and SpO_2_. The website collects patients’ health data from the apps, which are used during future hospital visits.

**User requirements for the mobile app and wearable device.**

**User requirements for the mobile app**

- App configuration should be easy to understand and use for all age groups.
- All menus pertinent to the current task should be displayed on the screen, and the menu structure should not be overly complicated.
- It should seamlessly integrate to wearable devices with smartphones.
- Biometric parameters of the patient heart rate, blood oxygen saturation (SpO_2_), and calorie consumption should be locally and securely stored in the smartphone.
- Step-by-step exercise guidance should be provided that reflect patients’ exercise capacity.
- Simple feedback of breathing difficulty during exercise should be included.
- Patient exercise history should be presented both graphically and numerically for easy peruse.
- Alarm function should be provided to alert the patient of critical health status (SpO_2_ and heart rate) during exercise.

**User requirements for wearable device**

- It should be easy to wear.
- It should display patient’s health status on the device screen.
- It should be easy to store and view measurements.

#### Apps

**Figure 4:**
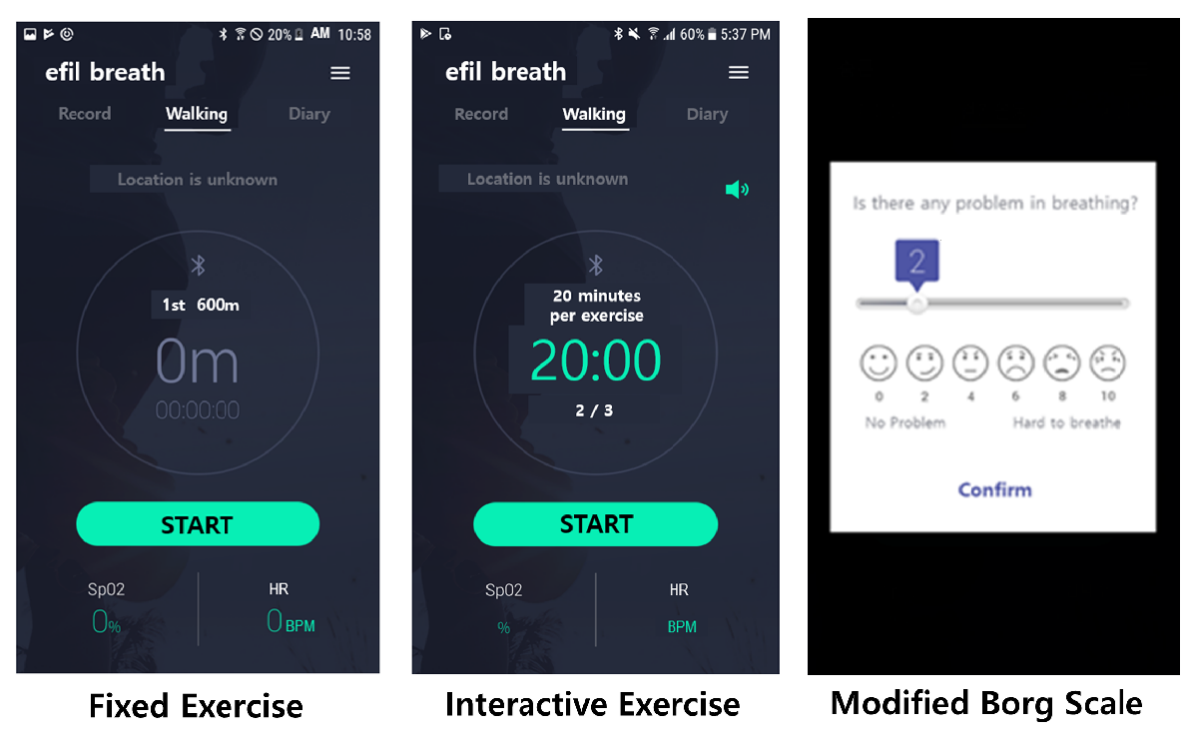
Fixed and interactive exercise regimens.

The goal of Stage 1 was to enable patients with COPD to maintain, at least, a minimum level of exercise per day (30 minutes). However, the feedback at the end of the pilot study expressed the desire that the app should offer longer exercise regimens with varying intensity; this was confirmed by the fact that 45% (5/11) of participants after 2 weeks and 55% (6/11) after 4 weeks stated that the exercise target was set too low (which might give the opportunity to set new targets as the PR continued). Therefore, at Stage 2, we developed 2 apps, one with a fixed exercise regimen, and the other with an interactive regimen for mobile phones running on the Android operating system (version 4.4.4 or above), as shown in Figure 4. Android was chosen as it was the most commonly used operating system in Korea.

The apps were linked to a wearable pulse oximeter via Bluetooth (version 4.0), and activity-related data (exercise compliance rate, heart rate, and SpO_2_) were sent to the monitoring website. Furthermore, a 6MWT was performed using the apps before initiating the exercise regimen.

**Figure 5:**
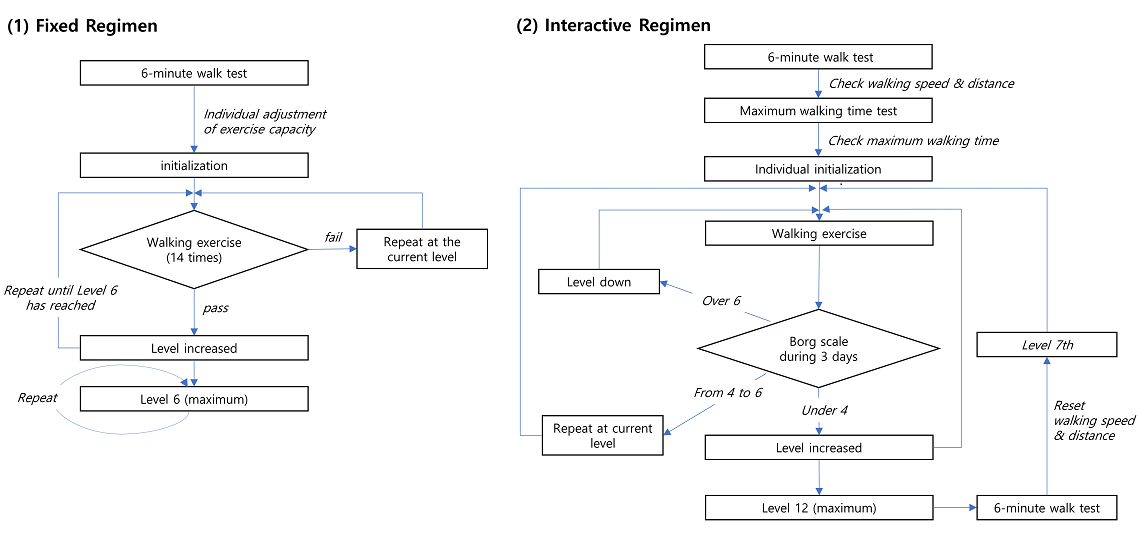
Walking exercise regimens: (1) fixed regimen and (2) interactive regimen.

Figure 5 shows the 2 exercise regimens used in the apps. The fixed regimen uses 6 levels of walking distance—600 m, 1200 m, 1800 m, 2400 m, 3000 m, and 3600 m. When the user achieves a fixed walking distance within a day and 14 times in total, the app increases the walking distance to the next level. The interactive regimen conforms to the exercise recommendations of the Consensus Document on Pulmonary Rehabilitation in Korea 2015 [29] and uses 12 levels [30]. The initial walking intensity is set to 80% of the maximum walking speed recorded in the 6MWT. Once initiated, a metronome in the app is used to help guide the walking speed of the patient. The level of exercise is then adjusted according to the modified Borg scale (0-10) [31] in the following manner. After the user has completed a walking session, the app with the interactive regimen asks to record the degree of breathing difficulty during exercise using the modified Borg scale. When a scale of ≤3 is recorded for 3 consecutive days, the exercise level goes up by 1, and when the scale persists ≥7, the level goes down by 1. In addition, when the final 12th level is reached, the patient is asked to perform a 6MWT, and the walking intensity is readjusted to an initial level of 7. The mobile phone vibrates when SpO_2_ falls <90% in both apps, prompting the patient to pause.

Furthermore, the apps provide guided resistance exercises that can be used at leisure by patients. The exercises feature audioguides and clickable links to external videos for further guidance. A simple exercise diary is available for both apps to help summarize daily exercise results such as calories burned, duration of exercise, distance walked, etc.

#### Central Patient Monitoring Website

The patient monitoring website acts as a central storage of records and history of the PR activities of patients. The secure database ensures that each participating hospital can only access its patient data. The patient health status is sent from the apps to the website in which the health care professionals view patient records such as patient PR compliance, heart rate, and SpO_2_ during exercise, and the 6MWT results.

**Figure 6:**
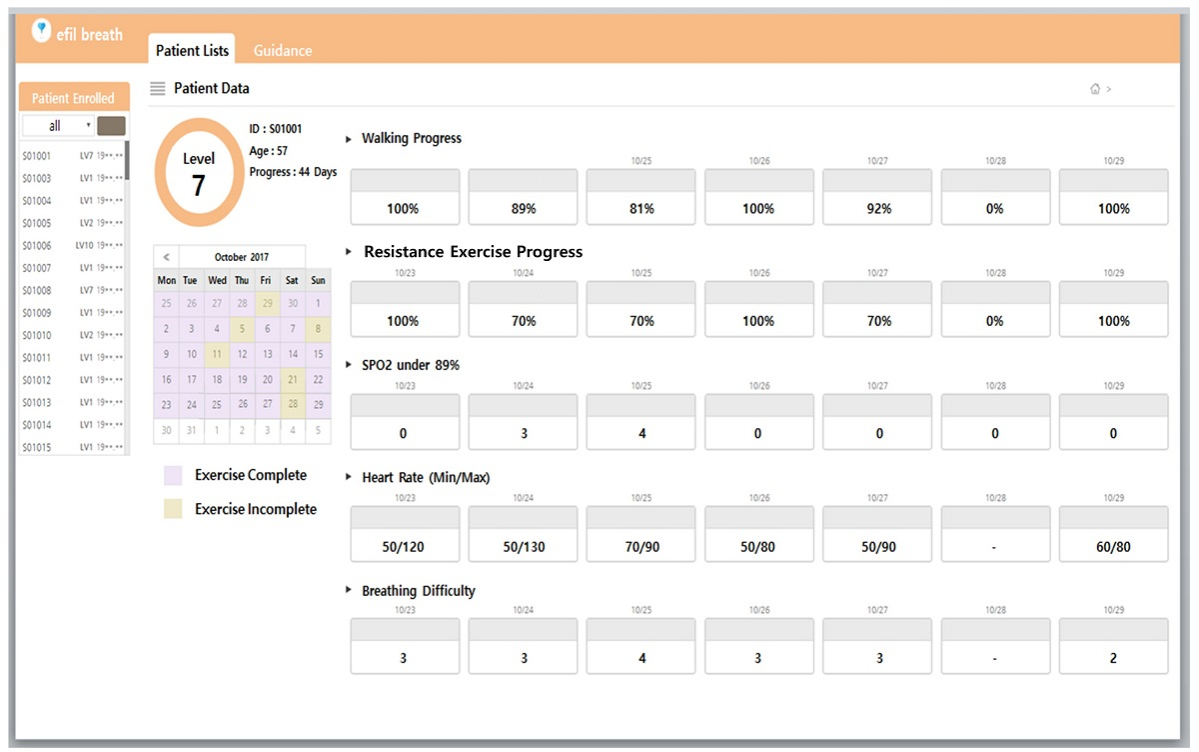
Patient pulmonary rehabilitation record.

The website provides a summary of the patient PR compliance of individual patients after enrollment. Figure 6 shows the PR records of a patient, such as their progress, heart rate, and distress. In addition, the website also enables health care professionals to view a list of patients with low SpO_2_ (<90%) and those experiencing breathing difficulties (Borg scale score ≥7) who need closer monitoring during the use of the app.

**Figure 7:**
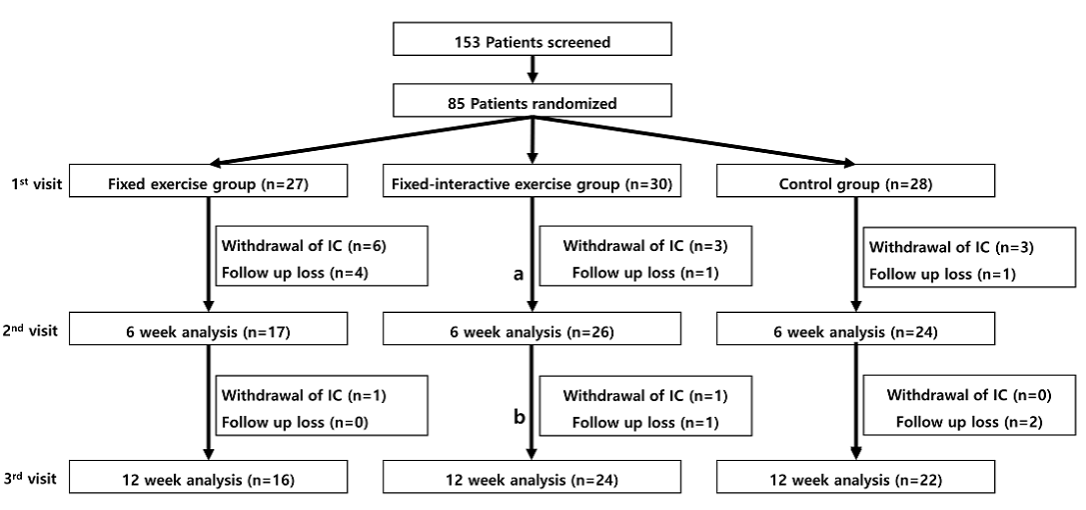
Study groups. IC: informed consent; a: Participants used fixed-regimen app; b: Participants used interactive-regimen app.

#### Participants’ Characteristics in the Clinical Trial

A total of 153 patients were screened, and 85 participants were randomized into 3 groups (Figure 7). Of these, 40 participants in the intervention groups and 22 in the control group completed the 12-week clinical trial. Table 1 presents the baseline characteristics. Overall, 82% of participants were males, and 29.4% were current smokers who smoked for 39 pack-years on average. Around 80% of participants were in Global Initiative for Chronic Obstructive Lung Disease Stage I or II. Notably, no significant difference was observed between the groups at the baseline such as age, exercise capacity, and lung function (Table 1).

**Table 1:** Participant characteristics (N=85).

Characteristics				Fixed group (n=27)		Fixed-Interactive group (n=30)		Control group (n=28)	*P* value
Age (years), mean (SD)				64 (8)		65 (7)		64 (8)	.86
Age (years), range				47-79		47-76		45-80	N/A^a^
**Gender, n (%)**									
	Male				23 (85)		26 (86)	21 (75)	.46
	Female				4 (15)		4 (13)	7 (25)	.46
Body mass index (kg/m^2^), mean (SD)				23.6 (3.7)		22.6 (3.0)		24.3 (3.9)	.19
Current smoker, n (%)				9 (33)		8 (27)		4 (29)	.85
Pack-year (years), mean (SD)				43 (25)		37 (19)		37 (17)	.57
6-min walking distance (m), mean (SD)				356 (98)		392 (84)		356 (84)	.21
FVC^b^ (L), mean (SD)				2.80 (0.87)		2.83 (0.84)		2.88 (0.66)	.95
FEV1^c^ (L), mean (SD)				1.52 (0.47)		1.52 (0.51)		1.43 (0.39)	.70
FEV1 (% predicted), mean (SD)				58.59 (15.75)		57.13 (16.74)		55.79 (15.48)	.81
FEV1/FVC (%), mean (SD)				55.04 (13.17)		56.73 (15.34)		52.67 (16.71)	.64
COPD assessment test score, mean (SD)				15.59 (7.84)		14.97 (8.48)		16.18 (16.71)	.87
**Modified Medical Research Council, n (%)**									
			0		0 (0)		0 (0)	1 (4)	.23
			1		11 (41)		16 (53)	6 (21))	.23
			2		12 (44)		11 (37)	15 (54)	.23
			3		3 (11)		3 (10)	6 (21)	.23
			4		1 (4)		0 (0)	0 (0)	.23
**Comorbidities, n (%)**									
			Yes		25 (93)		27 (90)	24 (89)	.91
			No		2 (7)		3(10)	3 (11)	.91
**Global Initiative for Chronic Obstructive Lung Disease stage, n (%)**									
		1			8 (31)		5 (17)	7 (28)	.93
		2			14 (54)		18 (62)	13 (52)	.93
		3			3 (12)		4 (14)	4 (16)	.93
		4			1 (4)		2 (7)	1 (4)	.93

^a^N/A: not applicable.

^b^FVC: forced vital capacity.

^c^FEV1: forced expiratory volume in one second.

**Figure 8:**
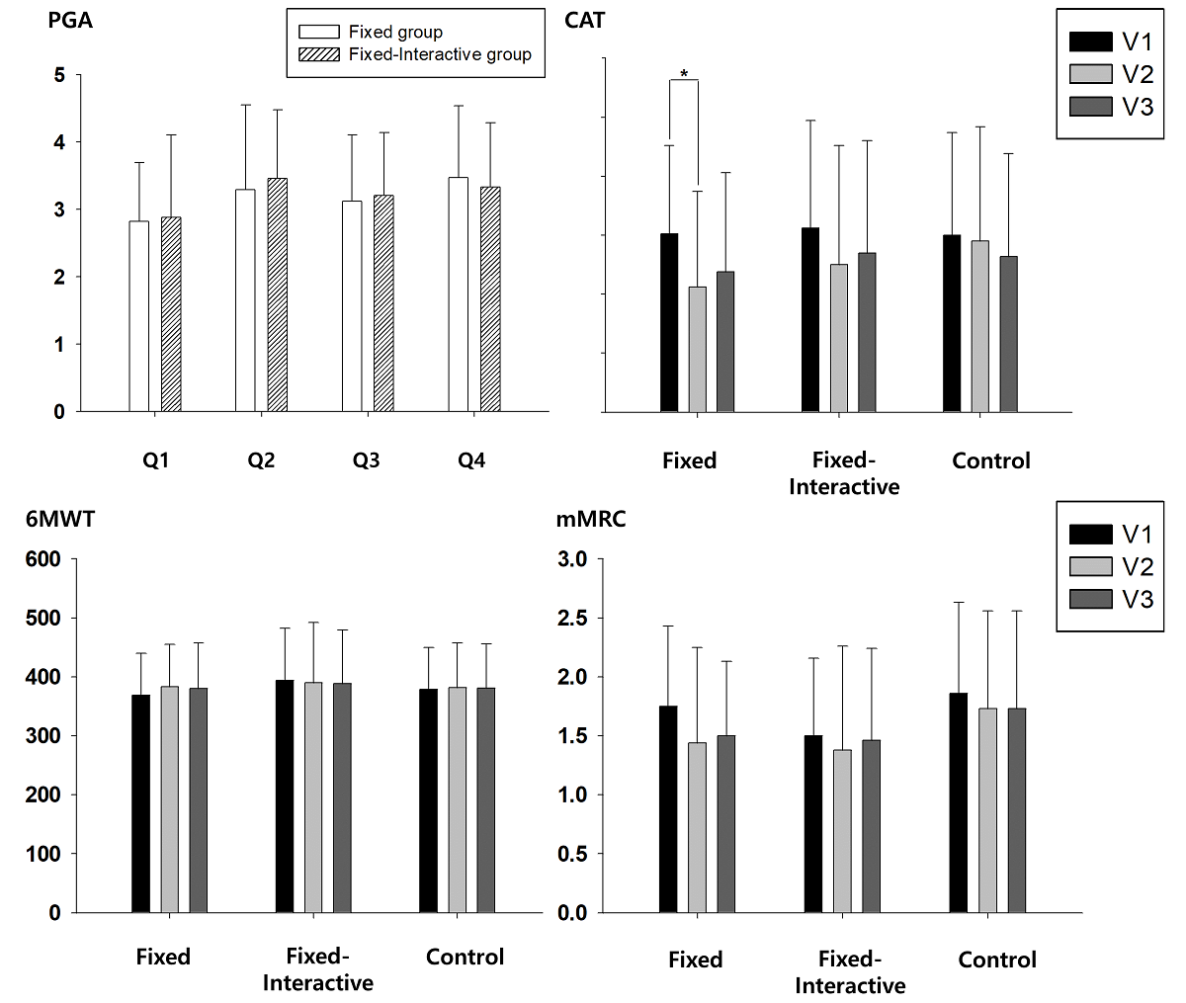
Respiratory function parameter changes and the patient global assessment of trial participants. PGA: patient global assessment; CAT: COPD assessment test; 6MWT: 6-minute walk test; mMRC: modified Medical Research Council; V1: baseline; V2: 6 weeks; V3: 12 weeks.

Regarding primary endpoints (Figure 8), CAT scores showed significant changes in the fixed group (*P*=.01) at V2, and some improvement was also observed in the fixed-interactive group (*P*=.06). The CAT scores at V3, however, did not show further improvement in both intervention groups. No significant change was observed in CAT scores in the control group throughout the trial. In addition, 6MWT and mMRC did not show statistically significant changes at V2 or V3 in all groups. PGA was measured at V3 for both fixed and fixed-interactive regimens, which showed a moderate level of satisfaction (min-max, 2.8-3.5) regarding the rehabilitation education content (Q2), helpfulness of content toward physical activity (Q3), and management of exercise and physical strength (Q4). The overall satisfaction of the apps (Q1) received an average of 2.8 points for both regimens.

## Discussion

### Principal Findings

Mobile PR for use by patients with COPD has great potential in improving health outcomes for patients, especially when it incorporates standardized guidelines for PR, and malleable exercise programs that can accommodate the diverse physical capacity of patients. Therefore, this study aimed to develop a mobile PR platform for COPD patients that offers an opportunity to observe the improvement or maintenance of daily physical capacity and QoL. The final technological aspects of this study consisted of (1) 2 mobile apps, one with a fixed exercise regimen and the other with flexible exercise regimen in accordance with the level of physical capacity; (2) a secure COPD patient management and monitoring server with a database used for better patient care. Moreover, our first randomized multicenter-based clinical trial in Korea demonstrated the benefits of applying mobile technology to the HBR of COPD patients.

Among respiratory function parameters, CAT scores showed significant improvement after 6 weeks in the fixed exercise group. As a matter of fact, both intervention groups underwent a fixed exercise regimen for the first 6 weeks. The statistical significance of improvement of CAT scores was high (*P*=.002) in the sum of patients in both groups after 6 weeks. Meanwhile, the majority (80%) of study participants in all groups had mild or moderate disease severity (Global Initiative for Chronic Obstructive Lung Disease stages I and II), and their 6MWT ranged 350-400 m at the baseline; this might explain why the 6MWT did not show significant improvement at V2 or V3. In addition, mMRC, a change-resistant outcome measure, did not show meaningful improvement in our trial, yet correlations between the measured CAT and mMRC changes were present in this study. Useful as it may, mMRC is more of a subjective value ranging from 0 to 4. Considering our patient population, the baseline respiratory function, exercise regimen, and study period, CAT may be the better outcome measure than mMRC in this type of study or even future trials [32].

Regarding the demographics of participants, we first speculated that the interactive regimen or app may present some difficulty in its use, owing to a relatively more complex interface. However, the difference in outcomes between the fixed and fixed-interactive regimens was interestingly largely insignificant. Despite the results, further refinement of the user interface would be necessary to assist users of our platform.

### Limitations

There are several limitations in the trial. First, study subjects were aware to which group they were allocated to during the study. We attempted to minimize further bias by blinding the person who obtained the primary endpoints or analyzed the data. The dropout rate in the intervention groups was noted to be slightly higher than expected, indicating that ongoing patient education and feedback would be required to maximize the benefits of adherence and better secure the merits of mobile PR. Hence, more studies are needed to direct the suitable outcome measure to assess the effectiveness of the utilization of appropriate mobile PR platforms, especially in use for patients with COPD.

### Conclusions

mHealth technology is on the verge of being sufficiently robust to be incorporated as an ancillary component of chronic disease management and rehabilitation. Its efficacy and relevance as an alternative or supplemental means for COPD rehabilitation can be strengthened by accumulating evidence in mobile rehabilitation programs and services; this will enable the prescription of flexible exercise regimens and commensurate with patients’ physical capacities. Moreover, a well-designed rehabilitation monitoring and management of patients with COPD will fill the gap left open by traditional center-based rehabilitation programs. Our *efil breath* is the first attempt in Korea at developing a comprehensive mHealth management platform for the rehabilitation of patients with COPD. Further research is required to study the long-term benefits of compatible mobile COPD rehabilitation services and to investigate the benefit of mobile-only COPD rehabilitation services for patients without access to local or regional clinical health care centers or health care providers.

## Appendix

Multimedia Appendix 1 CONSORT‐EHEALTH checklist (V 1.6.1).

## Abbreviations

CAT: COPD assessment test
COPD: chronic obstructive pulmonary disease
PGA: patient global assessment
PR: pulmonary rehabilitation
QoL: quality of life
HBR: home-based rehabilitation
